# Discovery of Distinctin-Like-Peptide-PH (DLP-PH) From the Skin Secretion of *Phyllomedusa hypochondrialis*, a Prototype of a Novel Family of Antimicrobial Peptide

**DOI:** 10.3389/fmicb.2018.00541

**Published:** 2018-03-23

**Authors:** Di Wu, Yitian Gao, Yining Tan, Yuzhang Liu, Lei Wang, Mei Zhou, Xinping Xi, Chengbang Ma, Olaf R. P. Bininda-Emonds, Tianbao Chen, Chris Shaw

**Affiliations:** ^1^Natural Drug Discovery Group, School of Pharmacy, Queen's University Belfast, Belfast, United Kingdom; ^2^AG Systematik und Evolutionsbiologie, IBU—Faculty V, Carl von Ossietzky University Oldenburg, Oldenburg, Germany

**Keywords:** frog skin secretion, molecular cloning, antimicrobial peptide, distinctin, anti-biofilm, anti-proliferation

## Abstract

Amphibian skin secretions are an important treasure house of bioactive antimicrobial peptides (AMPs). Despite having been the focus of decades of research in this context, investigations of phyllomedusine frogs continue to identify new AMPs from their skin secretions. In this study, the prototype of a novel family of AMP distinctin-like-peptide-PH (DLP-PH) was identified from the skin secretion of the otherwise well-studied Tiger-Legged Tree Frog *Phyllomedusa hypochondrialis* through cloning of its precursor-encoding cDNA from a skin secretion-derived cDNA library by a 3′-rapid amplification of cDNA ends (RACE) strategy. Subsequently, the mature peptide was isolated and characterized using reverse-phase HPLC and MS/MS fragmentation sequencing. DLP-PH adopted an α-helical conformation in membrane mimetic solution and demonstrated unique structural features with two distinct domains that differed markedly in their physiochemical properties. Chemically synthesized replicates of DLP-PH showed antimicrobial activity against planktonic bacterial and yeast cells, but more potent against *Escherichia coli* at 32 μg/mL. However, DLP-PH showed much weaker inhibitory activity against the growth of sessile cells in biofilms. In addition, DLP-PH exhibited anti-proliferative activity against human cancer cell lines, H157, and PC3, but with no major toxicity against normal human cell, HMEC-1. These combined properties make DLP-PH deserving further study as an antimicrobial agent and further investigations of its structure-activity relationship could provide valuable new insights into drug lead candidates for antimicrobial and/or anti-cancer purposes.

## Introduction

Not even a century after the discovery of penicillin marked a new era is the treatment of bacterial infectious agents, modern medicine is quickly reaching a crossroads with respect to the utility of antibiotics in health care. Two trends are especially apparent and worrisome in what is an evolutionary arms race. The first is the recent, rapid increase in the emergence of drug-resistant infectious agents (“superbugs”) caused, in part, through the inappropriate prescribing of conventional antibiotics (Poole, 2011). The second is the natural ability of diverse bacterial species to form biofilms. These biofilms, which are derived from the hydrated polymeric matrices of sessile bacterial cells, are potently resistant to conventional treatment because of their thick, slimy, and slippery coating and can thus cause persistent, serious infections. Progressive evidences on biofilms represent a natural defensive strategy on the part of the bacteria, in response to sub-inhibitory antibiotic doses (Karatan and Watnick, 2009). Whereas, recent efforts have been directed to some extent at modifying existing antibiotics to increase their potency (e.g., vancomycin; Okano et al., 2017), a useful alternative strategy might be the discovery and application of novel antibiotic compounds with a different mode of action to conventional antibiotics.

In this second context, antimicrobial peptides (AMPs) have attracted the attention of many researchers because of their numerous unique advantages, such as potent effects against a variety of pathogens, including many superbugs, their ability to suppress biofilm formation (Reffuveille et al., 2014; Pletzer and Hancock, 2016), and that their apparent primary mechanism of action (degradation of the microbial membrane) might prove to be more resistant to the development of antibiotic resistance. Moreover, in addition to their direct antimicrobial activities, AMPs have been recently discovered to possess beneficial, secondary actions such as mediating the immune response and enhancing both wound healing and angiogenesis (Samy et al., 2011; Habets and Brockhurst, 2012).

An important natural resource for AMPs, are the skin secretions of amphibians, which, in addition to AMPs, also contain a cocktail of other chemically complex bioactive molecules displaying a wide range of activities. Indeed, amphibians AMPs have attracted much attention over the past decades (Xu and Lai, 2015), with over one-third of the nearly 3000 naturally occurring AMPs listed in the Antimicrobial Peptide Database (http://aps.unmc.edu/AP) coming from amphibians and 90% of those from frogs and toads. Nevertheless, the potential of amphibian skin secretions as a source of AMPs and of the AMPs themselves as a source of novel antibiotics has barely been tapped. Many new amphibian AMPs remain to be discovered and the AMPs that are already known either possess potent antimicrobial activities that deserve further study or can be used as templates for computer-aided drug design to enhance their efficacy.

Within this latter context, we describe a novel AMP precursor that we identified from the skin secretion of *Phyllomedusa hypochondrialis*. The mature peptide, which we called distinctin-like-peptide-PH (DLP-PH), displayed a number of interesting characteristics deserving further investigation, including an evaluation of its bioactivities.

## Methods

### Secretion acquisition and maintenance of experimental specimens

Four adult specimens of *P. hypochondrialis* were obtained from a commercial source in Peru (PeruBiotech E.I.R.L., Lima, Santiago de Surco, Peru). The frogs were housed in a tropical frog vivarium at 25°C and 85% humidity under a 12 h/12 h day/night cycle and were fed multivitamin-loaded crickets three times per week. The skin secretions were sampled via the minimally invasive method of mild transdermal electrical stimulation (Tyler et al., 1992) after the frogs had been raised for 4 months under these conditions. For this study, all frogs have been raised for 23 months. The secretions were then washed into an ice-chilled glass beaker using distilled deionized water and lyophilised. In total, 29.7 mg of dry skin secretion were harvested. Secretion acquisition was performed under UK Animal (Scientific Procedures) Act 1986, project license PPL 2694 as issued by the Department of Health, Social Services and Public Safety, Northern Ireland. All procedures were vetted by the IACUC of Queen's University Belfast and approved on March 1, 2011.

### Construction of a skin secretion-derived cDNA library and “shotgun” cloning

Five milligrams of lyophilised *P. hypochondrialis* skin secretion were dissolved in 1 mL of cell lysis/mRNA binding buffer supplied in a Dynabeads Kit (Dynal Biotech, Merseyside, UK). The polyadenylated mRNA was isolated by using the magnetic oligo-dT beads in accordance with the manufacturer's instructions. The isolated mRNAs were then reverse-transcribed to construct a cDNA library using a SMART-RACE Kit (Clontech, Palo Alto, CA, USA) and the 3'-RACE reactions to acquire full-length prepropeptide nucleic acid sequence data. Potential AMPs were then amplified from this library through the combination of the nested universal primer (5′-AAGCAGTGGTATCAACGCAGAGT−3′) that was supplied with the kit with a degenerate primer (5′-ACTTTCYGAWTTRYAAGMCCAAABATG-3′) that we designed to bind to a highly-conserved segment of the 5′-untranslated region of bioactive peptide cDNAs from phyllomedusine frogs (Wechselberger et al., 1998; Pierre et al., 2000). The RACE products were purified using a Cycle-Pure Kit (Omega Bio-Tek, Norcross, GA, USA) and cloned into a pGEM-T vector (Promega Corporation, Southampton, UK). Sequencing reactions were performed using a BigDye Terminator Sequencing Kit (Applied Biosystems, Foster City, CA, USA) in combination with an ABI 3100 automated capillary sequencer (Applied Biosystems, Foster City, CA, USA).

### Identification and primary structure analysis of mature peptide in crude skin secretion

Another five milligrams of the lyophiled skin secretion of *P. hypochondrialis* were dissolved in 0.5 mL of 0.05/99/95 (v/v) trifluoroacetic acid (TFA)/water and centrifuged for clarification. The supernatant was then analyzed using a gradient RP-HPLC system (Waters, Milford, MA, USA) fitted with an analytical column (Aeris PEPTIDE XB, C18, 5 μm, 10.0 × 250 mm, Phenomenex, Macclesfield, Cheshire, UK). Contents were eluted from the column using a linear gradient composed of 0.05/99.95 (v/v) TFA/water to 0.05/19.95/80.00 (v/v/v) TFA/water/acetonitrile over 240 min at a flow rate of 1 mL/min. The effluents were collected at 1-min intervals and their UV absorbance were monitored at both 214 and 280 nm simultaneously. Samples from each fraction were subsequently analyzed by MALDI-TOF MS (Voyager DE, PerSeptive Biosystems, Foster City, CA, USA) using α-cyano-4-hydroxycinnamic acid as the matrix. The fraction that contained a peptide with a molecular mass coincident with the mass of the mature peptide deduced from the cloned precursor-encoding cDNA was infused onto an LCQ-Fleet ion-trap MS (Thermo Fisher Scientific, San Francisco, CA, USA) for fragmentation sequencing.

### Solid-phase peptide synthesis

Following the unequivocal confirmation of the primary structure of the mature peptide in the preceding step, the native peptide as well as three truncated versions of it (the N-terminal domain, the C-terminal domain, and a slightly extended version of the latter) were chemically synthesized via solid-phase Fmoc chemistry using a Tribute automated synthesizer (Protein Technologies, USA). Corresponding Wang-resins, protected amino acids, N-methylmorpholine (NMM), piperidine, 2-(1H-benzotriazol-1-yl)-1,1,3,3-tetramethyluronium hexafluorophosphate (HBTU), N,N-dimethylformamide (DMF), and dichloromethane (DCM) were used during the synthesis. The TFA/triisopropylsilane (TIPS)/water cocktail was used to cleave the peptide from the resin and to deprotect the side-chains. All peptides were analyzed and purified using RP-HPLC. Both molecular masses from MALDI-TOF and MS/MS fragmentation profiles were also employed to determine the purity and authenticity of the respective structures.

### Determination and visualization of peptide secondary structures

In the first instance, secondary structures of the synthesized peptides were determined via circular dichroism (CD) using a JASCO J-815 CD spectrometer (Jasco, Essex, UK). The peptides were dissolved initially in 10 mM ammonium acetate and 50% TFE in 10 mM ammonium acetate to reach a concentration of 100 μM and then added into a 1-mm high-precision quartz cell (Hellma Analytics, Essex, UK). CD spectra were obtained at 20°C from 190 to 250 nm at a scanning speed of 100 nm/min with 1 nm bandwidth and 0.5 nm data pitch.

Additionally, we used several modeling methods to further elucidate secondary structures as well as to investigate possible mechanisms of action of the peptides. Helical Wheel Projection (http://rzlab.ucr.edu/scripts/wheel/wheel.cgi) was used to visualize the α-helical peptide and the I-TASSER online server (Yang et al., 2015) was employed to predict the secondary structures of the native peptide and its derivatives and to infer their 3-D structural models. The overall quality of these models were quantified by Ramachandran plots using RAMPAGE (Lovell et al., 2003) and by z-scores using ProSA (Wiederstein and Sippl, 2007). The physiochemical parameters of the AMPs were predicted using HeliQuest (Gautier et al., 2008).

As DLP-PH was more effective against the growth of *E. coli*, we aimed to simulate the antimicrobial mechanism using the *E. coli* membrane model (Pandit and Klauda, 2012), which has been fit to experimentally determined results and consists of six different lipids, was reconstructed through CHARMM-GUI (Jo et al., 2008). We then adopted the water-removed model to simulate AMP-membrane interaction. Molecular docking was performed using AutoDock Tools and AutoDock Vina (Trott and Olson, 2010). The aforementioned water-removed membrane model was used as the receptor molecule, the absent polar hydrogens were added to this molecule through AutoDock Tools and then saved as a formatted pdbqt file. The grid box was set to cover the outer leaflet with the center in 0, 0, 14 (x, y, z); and its size was set to 69 for each of the three axes, the optimal box size 69 for DLP-PH as determined by the eBoxSize script (Feinstein and Brylinski, 2015). The DLP-PH model file was input to AutoDock Tools as the ligand molecule. The Gasteiger charges were added and the non-polar hydrogens were merged automatically. Then the missing polar hydrogens were added, the chemical bonds torsions were adjusted and the molecule was output to a pdbqt formatted file. Exhaustiveness was set to 20 given that the volume of the search space was larger than 27000 Å^3^. All the information was written into a configuration file and was calculated by AutoDock Vina. The calculated molecular docking result with the best affinity score was rendered with the PyMol (PyMOL Molecular Graphics System, Version 1.8 Schrödinger, LLC).

### Antimicrobial susceptibility assays

The antimicrobial activities of the synthetic peptides were assayed against both planktonic microbial cells as well as sessile cells in biofilms. In the former case, we quantified both the minimal inhibitory (MIC) and minimal bactericidal concentrations (MBC) against planktonic cells of the gram-positive bacterium *Staphylococcus aureus* (NCTC 10788), the gram-negative bacteria *Escherichia coli* (NCTC 10418) and *Pseudomonas aeruginosa* (ATCC 27853), and the yeast *Candida albicans* (NCYC 1467) using the micro broth dilution method (Gao et al., 2016; Wu et al., 2016). The peptides were dissolved in physiological phosphate buffered saline (PBS) to prepare a series of 100-fold stock solutions via 2-fold dilutions, where 2 μL of each peptide solution was mixed with 198 μL of diluted microbial culture to achieve a range of final concentrations from 512 to 1 μg/mL. Similarly, a divalent cation supplemented medium (20 mg/L Mg^2+^ and 10 mg/L Ca^2+^) was also used to determine the MICs in the presence of cations. For all assays, MICs were defined as the lowest concentration of peptides that resulted in the absorbance less than 0.1 in the 96-well plates whereas MBCs were defined as the lowest concentration that showed no colony growth on MHA plates.

Susceptibility assays against sessile cells in biofilms were conducted by quantifying the minimal biofilm inhibition (MBIC) and the minimal biofilm eradication concentrations (MBEC) as well as the biofilm initial attachment inhibition against all the strains. MBIC and MBEC testing was performed, with minor modifications, according to Knezevic and Petrovic (2008) and Sabaeifard et al. (2014), using the colourimetric indicator 2,3,5,-triphenyl tetrazolium chloride (TTC) to estimate microbial cell viabilities. *C. albicans* was cultured in RPMI-1640 and the other strains were cultured in MHB with 2% glucose, respectively. Overnight cultures were washed once with pre-warmed PBS and diluted with fresh medium to 10^7^ CFU/mL. For the MBIC assay, the peptide stock solutions were prepared in the same way with the same concentration range as for the MIC assays and then incubated at 37°C for 24 h. Thereafter, the plates were washed twice with PBS followed by the addition of 200 μL fresh MHB with 2% glucose (or RPMI-1640 for *C. albicans*) and 50 μl 1% TTC (g/v) solution per well. After 5 h incubation, 200 μL of the supernatant from each well were transferred to a new plate and its absorbance at 470 nm was determined using a Synergy HT plate reader (BioTek, Winooski, VT, USA). For the MBEC assay, 200 μL of the same diluted inoculum was dispersed into each well in a flat-bottomed 96-well plate for 48 h to form mature biofilms. Following sufficient time, the plates were washed three times with PBS to remove the planktonic cells and incubated with the same concentration range of peptide solutions as above at 37°C for 24 h. Thereafter, each well was washed, refilled with fresh medium, stained with TTC, and incubated for a further 5 h. Absorbance was measured in the same way as with the MBIC assays. MBICs and MBECs were determined as the lowest concentrations at which no colourimetric metabolites were formed by the bacteria.

The biofilm initial attachment (IA) inhibition assay was performed using *P. aeruginosa*, a strong biofilm-producing bacterium, using a slightly modified version of the method of Zhang et al. (2016). Briefly, different concentrations of the peptide solutions and diluted bacterial inoculum were loaded in the same way as with the MBIC assays, but with an incubation time of only 1 h and also without agitation to facilitate bacterial binding. Thereafter, the wells were washed with PBS, fixed with methanol, air-dried and stained with 0.1% (w/v) crystal violet, and washed with tap water before being air-dried. Finally, the crystal violet was solubilized in 33% acetic acid and the absorbance at 550 nm was measured in a plate reader to calculate the IC50 of biofilm initial attachment inhibition.

### Cytoplasmic materials leakage assay

As an indicator of the potential lysis of the microbial cell membranes in the presence of the peptides, we performed a cytoplasmic material leakage assay (Sahu et al., 2009; Samanta et al., 2013). Briefly, overnight microbial cultures of *S. aureus, E. coli*, and *C. albicans* were washed with pre-warmed PBS and diluted to 5 × 10^5^ CFU/mL in PBS. The synthesized peptides to be tested were dissolved in PBS and 2-fold diluted as described above to achieve a final concentration range from 512 to 1 μg/mL. Triton-X 100 (0.2%) in PBS was used as a positive control, with 100 μL of different concentrations of the peptide in PBS (512 to 1 μg/mL) being used as blank controls. The plates were incubated at 37°C for 2 h before the contents in each well were filtered through 0.22-μm syringe filters (Sigma-Aldrich, St. Louis, MO, USA) into new plates. A volume of 100 μL of the supernatant was transferred into a new 96-well plate and the absorbance at 260 nm was measured using a plate reader.

### Haemolysis assay

We examined for potential hemolytic activity of DLP-PH and its derivatives using a 4% suspension of horse red blood cells (TCS Biosciences, Botolph Claydon, Buckingham, UK) in physiological PBS. Each peptide was tested within the concentration range of 1 to 512 μg/mL (see above), with each peptide concentration being incubated with the red blood cell suspension at 37°C for 2 h. PBS solution and an equal volume of PBS solution containing 2% of Triton X-100 (Sigma-Aldrich, St. Louis, MO, USA), a non-ionic detergent, were used as negative and positive controls, respectively. After 2 h, each peptide-cell mixture was centrifuged and 200 μL of supernatant were added to each well of a 96-well microtiter plate. The degree of lysis of the red blood cells was assessed by measuring the absorbance at 550 nm using a microplate reader.

### Assessment of anti-proliferation effect and cytotoxicity on human cancer and normal cell lines

Possible anti-cancer activities of the novel peptide were investigated against a range of five human cancer cells lines: human prostate carcinoma cell line PC-3 (ATCC-CRL-1435) and human non-small cell lung cancer cell line H-157 (ATCC-CRL-5802) were cultured in RPMI-1640 medium (Gibco, Invitrogen, Paisley, UK) with 10% fetal bovine serum (FBS) (Sigma-Aldrich, St. Louis, MO, USA) and 1% Penicillin-Streptomycin (PS) (Invitrogen, Paisley, UK), whereas human breast melanoma cell line MDA-MB-435s (ATCC-HTB-129), human breast adenocarcinoma cell line MCF-7 (ATCC-HTB-22), and human neuronal glioblastoma cell line U251MG (ECACC-09063001) were cultured in DMEM medium (Invitrogen, Paisley, UK) with 10% FBS and 1% PS. As a control, human microvessel endothelial cell line HMEC-1 (ATCC-CRL-3243), which was cultured in 10% FBS, 10 ng/mL epidermal growth factor (EGF) (Invitrogen, Paisley, UK), 10 mM L-glutamine (Invitrogen, Paisley, UK) and 1% PS loaded MCDB131 medium (Invitrogen, Paisley, UK), was used to evaluate the cytotoxicity of the synthetic peptides on normal human cell line.

Cell antiproliferative activity was assessed using the MTT cell viability assay. Briefly, the cells were counted and seeded in 96-well plates at a density of 5000 cells per well for 24 h. Thereafter, the cells were synchronized by treating them with serum-free medium for a further 12 h after which they were incubated for 24 h with the peptide dissolved in serum-free medium over a concentration range of 419.7 to 4.197 × 10^−3^ μg/mL. After the incubation period, 10 μL of 5 mg/mL MTT solution (Sigma-Aldrich, St. Louis, MO, USA) were added to each well and incubated for a further 4 h. All cells were cultured in an incubator with a humidified environment with 5% CO_2_ at 37°C. Finally, the supernatant in each well was removed and replaced with 100 μL dimethylsulfoxide (DMSO) and the absorbance of each well at 570 nm being determined using a plate reader.

Cell cytotoxicity activity was assessed using the LDH assay (Pierce LDH cytotoxicity assay kit, Thermo Scientific), and the experiment followed the instruction provided with the kit. The cells were seeded in a 96-well plate as described above and synchronized for a further 4 h. Afterwards, the cells were treated with the same concentration range of DLP-PH as used in the MTT assay and incubated for 45 min. Lysis solution and ddH_2_O were applied as positive and negative control, respectively. After incubation, 50 μL of each sample medium were transferred into another 96-well plate and mixed with 50 μL of LDH reaction mixture. Each sample was further mixed with 50 μL of stop solution after a 30 min incubation period with the absence of light at room temperature. The LDH activity was measured by the absorbance at 490 nm, which subtracted the absorbance at 680 nm.

### Statistical analyses

For all assays, all peptide concentrations and controls were tested in three independent experiments of five replicates each. The statistical analyses employed *t*-test and one-way ANOVAs, with the statistical significance of the differences being indicated as ^*^(*p* < 0.05), ^**^(*p* < 0.01), and ^***^(*p* < 0.001). The dose-response curves were constructed using a “best-fit” algorithm and HC_50_ and IC_50_ values were calculated through the data-analysis package in GraphPad Prism 6 (GraphPad Software, La Jolla, CA, USA).

## Results

### Identification and characterization of a DLP-PH precursor cDNA from a skin secretion-derived cDNA library

**Figure 1:**
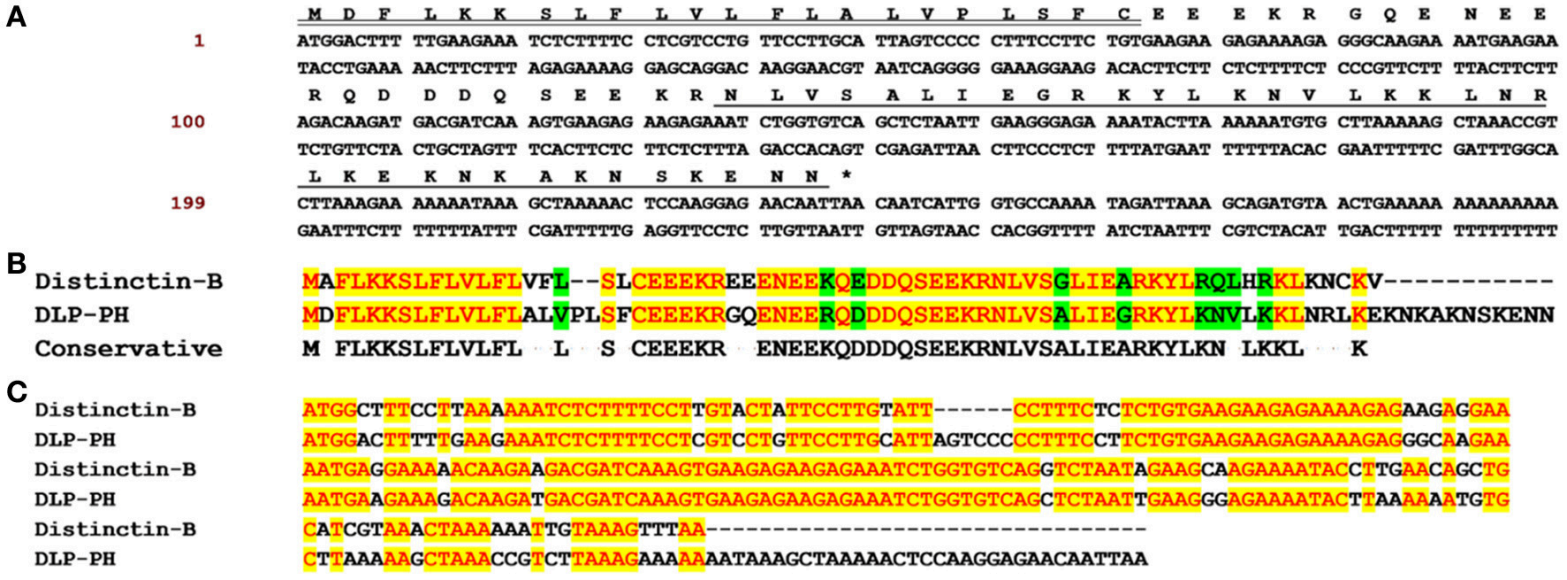
The biosynthetic precursor of DLP-PH and the comparison with Distinctin-B. **(A)** Nucleotide and translated open-reading frame amino acid sequence of a cDNA encoding the DLP-PH precursor cloned from a *P. hypochondrialis* skin secretion library. The predicted mature peptide is single underlined, the signal peptide is double-underlined and the stop codon is indicated by an asterisk. **(B)** The alignment of amino acid sequences of distinctin-B and DLP-PH precursors. **(C)** The alignment of corresponding precursor-encoding cDNA sequences. Conserved residues/bases are indicated in red with a yellow highlight. Similar residues are highlighted in green.

Through shotgun cloning of the *P. hypochondrialis* skin secretion derived cDNA library, we consistently retrieved a cDNA encoding the precursor of a novel peptide (DLP-PH) with an open reading frame of 80 amino-acid residues, including a signal peptide terminating in a cysteine residue; an acidic amino-acid residue-rich spacer peptide; and a predicted putative mature peptide of 36 amino-acid residues. The predicted mature peptide appeared after a lysine-arginine (KR) motif, which is a typical propeptide convertase cleavage processing site (Figure 1A). The nucleotide sequence of the entire cDNA encoding DLP-PH has been deposited in the EMBL sequence database under the accession number LT718215. An NCBI BLAST analysis, showed that the DLP-PH precursor exhibited a high degree of structural identity with another distinctin-like peptide precursor from *Phyllomedusa azurea*, including an identical mature peptide region and highly-conserved signal/spacer peptide regions (Accession No.: Q17UZ0). Additionally, the DLP-PH peptide precursor demonstrated strong similarity to the B-chain peptide (distinctin-B) precursor of the heterodimeric AMP distinctin from *Phyllomedusa distincta* (Batista et al., 2001; Evaristo et al., 2013) along most of the entire precursor-encoding cDNA of the latter. Noticeable dissimilarity only arose in the C-terminal domain of the mature peptides, with DLP-PH also being much longer than distinctin-B (Figures 1B,C).

MALDI-TOF mass spectrometry analyses of the fractionated skin-secretion samples from RP-HPLC (Figure S1A) identified a peptide coincident in molecular mass to that of the mature peptide predicted from the cloned cDNA (4196.9) in fraction #118. MS/MS fragmentation sequencing of a sample of this fraction (Figure S1B) confirmed the primary structure of the mature peptide.

### Prediction of secondary structure and physiochemical properties

The z-score of −1.34 for the 3D model of DLP-PH predicted using I-TASSER is within the range of scores typically found for protein/peptide native folds identified by NMR with similar size (Figure S2A). Moreover, assessment of stereo-chemical backbone of the DLP-PH model via the Phi and Psi dihedral angles of the Ramachandran plot showed that all residues were in the favored regions (Figure S2B). The model revealed that the peptide should adopt an α-helical conformation, which was verified by CD assays. Whereas, the mature peptide existed as a random coil in aqueous solution, it did indeed form a typical α-helical structure with negative bands at 208, 222 nm and a positive band at 190 nm in the membrane-mimetic TFE solution (Figure S2C).

**Figure 2:**
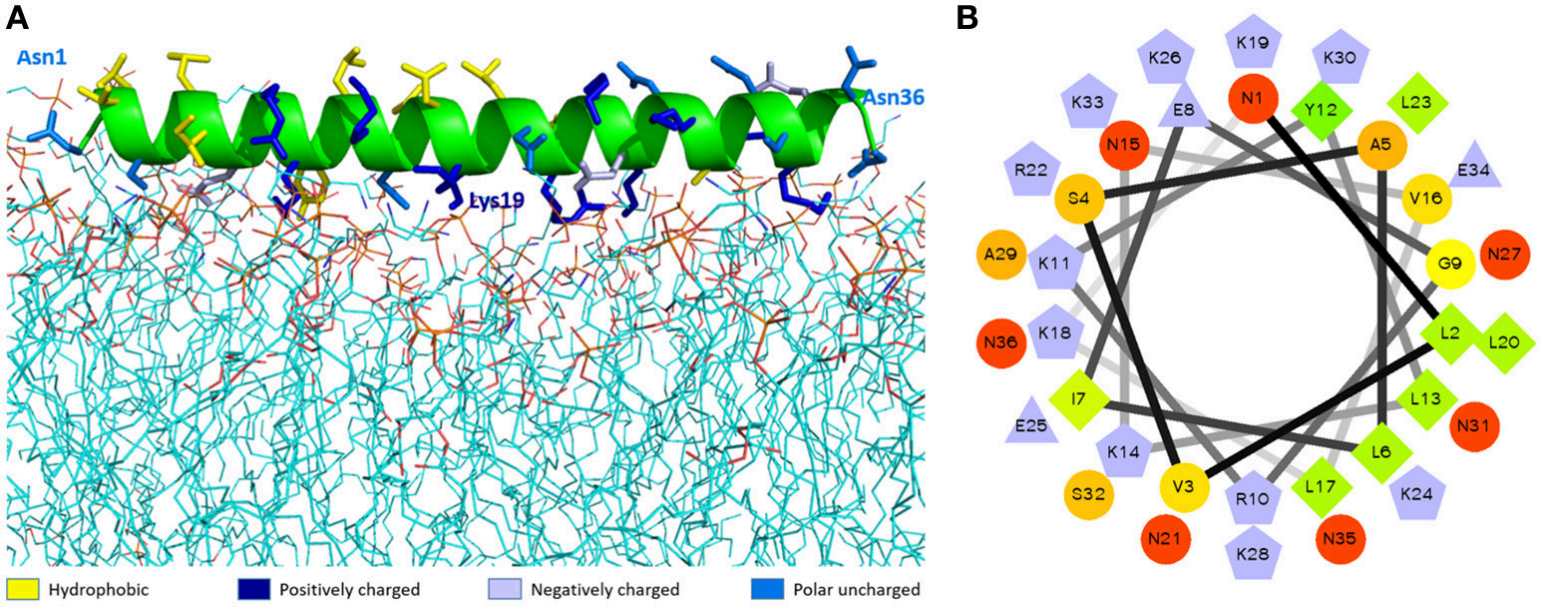
The molecular modeling of DLP-PH structures. **(A)** Theoretical docking study of DLP-PH with *E. coli* model membrane. The secondary structure of DLP-PH is showing in green cartoon, the main chain is hided and side chains are representing in sticks with different colors (designated in the figure according to the properties of the residues). The phospholipids are presenting in lines with carbon atoms in cyan, oxygen atoms in red, nitrogen atoms in blue, and phosphorus atoms in orange. **(B)** Helical wheel projection of DLP-PH with the N-terminal domain in the inner circle and the C-terminal domain in the outer circle.

The best molecular docking model between DLP-PH and the model of the *E. coli* cytoplasmic membrane showed a binding affinity of −3.5 kcal/mol, with DLP-PH bonding to the hydrophilic heads of the phospholipid molecules. Parallel to this, most of the cationic charged residues (Lys and Arg) of DLP-PH were predicted to face the cell membrane through electrostatic attraction (Figure 2A). Both the binding model and the DLP-PH helical wheel projection plot of DLP-PH (Figure 2B) indicated that the N-terminal (inner circle in Figure 2B) and C-terminal domains of the mature peptide (outer circle) possess distinctly different physiochemical properties, with the former being amphipathic and the latter hydrophilic. We confirmed this by synthesizing two additional peptides corresponding to the two domains of the full peptide (DLP-PHn and DLP-PHc, respectively) (Table 1). Thus, whereas the entire peptide is hydrophilic (H = −0.031) and cationic (+8) nature because of the high proportion of polar residues, DLP-PHn is amphipathic (μH = 0.487) with a high hydrophobicity (H = 0.358) and an outstanding hydrophobic face (-YAVGLLLL-) and DLP-PHc with its comparative lack of non-polar residues shows poor hydrophobicity (H = −0.480) and amphipathicity (μH = 0.158) (Table 2). A third peptide, DLP-PHt, was designed by truncating the native peptide at Asn^15^ according to that portion of DLP-PH that shows distinctly reduced homology with the B-chain of distinctin (Lys^14^ to Asn^36^) while avoiding Lys^14^, which might be enzymatically sensitive. Compared with DLP-PHc, DLP-PHt displayed a higher hydrophobicity (H = −0.283) and amphipathicity (μH = 0.270), but with values that were still distinctly lower than those for DLP-PH and DLP-PHn.

**Table 1:** Sequences of DLP-PH, its derivatives, distinctin, and distinctin-B.

**Peptide**	**Sequence**
DLP-PH	NLVSALIEGRKYLKNVLKKLNRLKEKNKAKNSKENN
DLP-PHn	NLVSALIEGRKYLKNVLK
DLP-PHc	KLNRLKEKNKAKNSKENN
DLP-PHt	NVLKKLNRLKEKNKAKNSKENN
Distinctin-B	NLVSGLIEARKYLEQLHRKLKNCKV
Distinctin	ENREVPPGFTALIKTLRKCKII (A) | NLVSGLIEARKYLEQLHRKLKNCKV (B)

**Table 2:** Physiochemical properties of DLP-PH and its derivatives.

**Region**	**Hydrophobicity <H>**	**Hydrophobic moment <μH>**	**Polar residues and G <n>**	**Non-polar residues <n>**	**Net charge <z>**
DLP-PH	−0.031	0.313	24	12	+8
DLP-PHn	0.358	0.487	9	9	+3
DLP-PHc	−0.420	0.158	15	3	+5
DLP-PHt	−0.283	0.270	17	5	+6

### Antimicrobial activities

**Figure 3:**
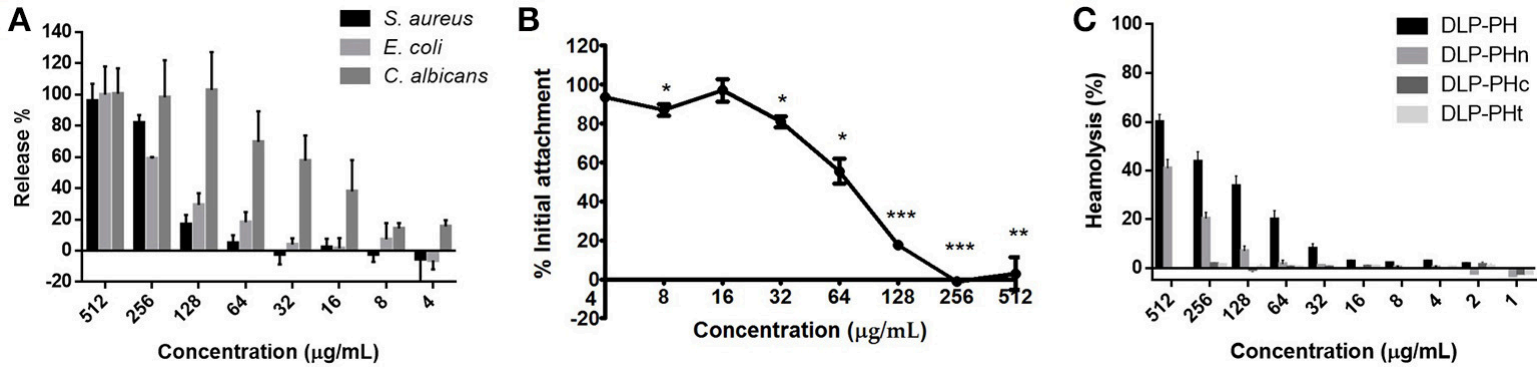
**(A)** Effect of DLP-PH on cytoplasmic material release of *S. aureus, E. coli*, and *C. albicans* at 260 nm. The error bar represents the standard error for three repeats (ANOVA, *P* < 0.05). **(B)** Effect of DLP-PH on the *P. aeruginosa* biofilm initial attachment. The error bar represents the standard error for three repeats. The significance is given as ^*^p < 0.05; ^**^p < 0.01; ^***^p < 0.001. **(C)** Relative haemolysis of DLP-PH and its deviates on horse red blood cells. The 100% haemolysis was defined using haemolytic effect induced by 1% Triton X-100 (ANOVA, *P* < 0.05).

The naturally occurring peptide DLP-PH exhibited activity against planktonic cells of all four tested microorganisms, albeit with noticeably less potency against the gram-positive bacterium *S. aureus* or in the presence of divalent cations (Table 3). Most of this activity appears to derive from the N-terminal domain of the peptide, with DLP-PHc and DLP-PHt being devoid of effective antimicrobial effects. Only DLP-PHn showed any noteworthy efficacy, which was at least 2-fold more effective than that of DLP-PH in the case of *S. aureus*. [However, because the molecular mass of DLP-PHn (2059 Da) is nearly half of that of DLP-PH (4197 Da), their MICs against *S. aureus* were quite similar when viewed in terms of molarity: 62.2 vs. 61.0 μM, respectively]. The efficacy of DLP-PH against sessile cells of all strains in biofilm was strongly reduced compared to the analogous values against planktonic cells (4- and 8-fold reduced, respectively; Table 4). The inhibition of biofilm initial attachment (IA) against *P. aeruginosa* showed an IA-IC_50_ value of 96 μg/mL (22.8 μM) (Figure 3B).

**Table 3:** Antimicrobial activity of DLP-PH and its derivatives against planktonic cells of four reference microorganisms.

	***S. aureus***		***E. coli***		***P. aeruginosa***		***C. albicans***	
	**MIC**	**MBC**	**MIC**	**MBC**	**MIC**	**MBC**	**MIC**	**MBC**
DLP-PH	256	512	32(128)	32	64(>512)	128	64	64
DLP-PHn	128	128	128	128	>512	>512	64	64
DLP-PHc	>512	>512	>512	>512	>512	>512	512	>512
DLP-PHt	512	>512	>512	>512	>512	>512	256	256

*Unit: μg/mL; values in brackets were the MICs tested in divalent cation supplemented medium*.

**Table 4:** Anti-biofilm activity of DLP-PH against all tested microorganisms.

**Microorganisms**	**MBIC**	**MBEC**
*S. aureus*	>512 (>122.0)	>512 (>122.0)
*E. coli*	128 (30.5)	256 (61.0)
*P. aeruginosa*	512 (122.0)	512 (122.0)
*C. albicans*	256 (61.0)	512 (122.0)

*μg/mL, molarity was calculated and shown in the brackets*.

The cytoplasmic materials release experiment revealed that DLP-PH lysed nearly 100% of the microbial cells at high concentrations of peptide (512 μg/mL; Figure 3A). Effects at lower concentrations were organism-specific. Thus, whereas strong cell lysis was also observed at the MIC of DLP-PH against *S. aureus* (256 μg/mL), barely any cells of *E. coli* were lysed at its MIC of 32 μg/mL. By contrast, DLP-PH continued to result in >50% cell lysis of *C. albicans* even at concentrations well-below its MIC (64 μg/mL) against this organism.

### Bioactivity of DLP-PH on mammalian cells

Only DLP-PH and DLP-PHn showed any haemolytic activity against horse blood red cells, but 100% haemolysis was never observed even at the highest tested concentration of 512 μg/mL. The remaining designed peptides, DLP-PHc and DLP-PHt, showed no haemolytic activity whatsoever, thereby mirroring their weak antimicrobial activities (Figure 3C).

**Figure 4:**
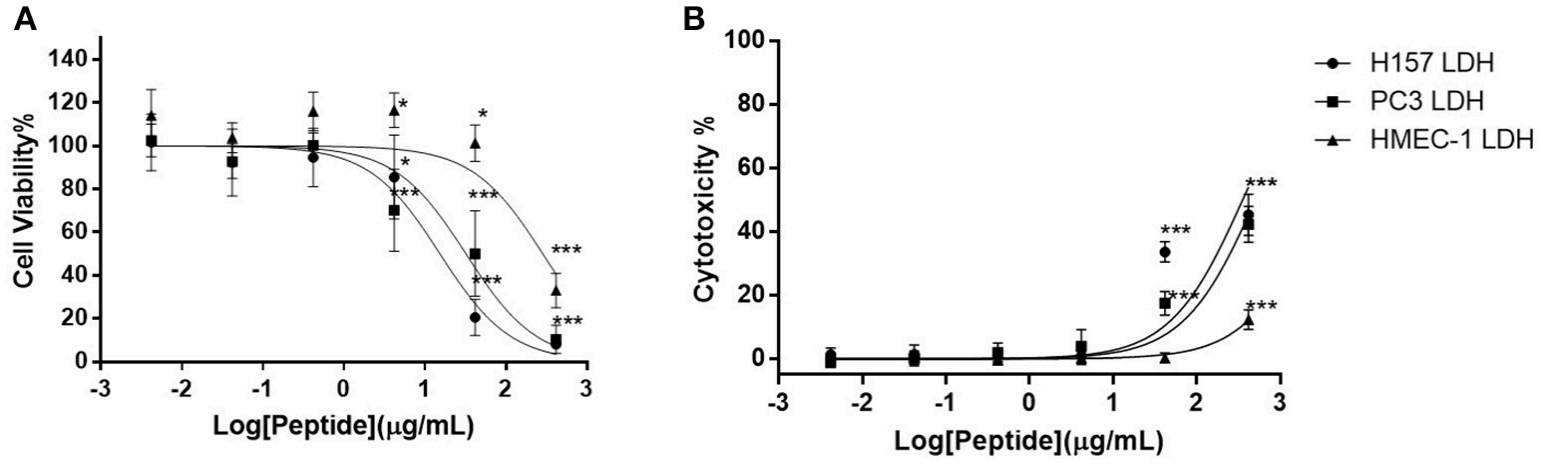
**(A)** The anti-proliferation effect and **(B)** cytotoxicity of DLP-PH on human cancer cell lines, H-157 and PC-3, and the normal human endothelial cell line, HMEC-1 within the concentration range of 419.7 to 4.197 × 10^−3^ μg/mL. The error bar represents the standard error for three repeats. The significance is given as ^*^p < 0.05; ^**^p < 0.01; ^***^p < 0.001.

Of the five different cancer cell lines used to screen the anti-proliferation activity of DLP-PH, evident activity (defined as a growth inhibition concentration ≥4.197 μM) was only shown against the two cell lines H-157 and PC-3, with IC_50_ values 15.41 and 32.25 μg/mL, respectively (Figure 4A). By contrast, DLP-PH showed significant cytotoxicity against the normal human cell line HMEC-1 only at the highest concentration tested (419.7 μg/mL), with the IC_50_ of 287.2 μg/mL also being much higher than the corresponding values for the two cancer lines above (Figure 4A). The LDH assay confirmed that DLP-PH resulted in low degree of cytotoxicity on mammalian cells, especially HMEC-1, while it exhibited more toxic effect on cancer cell lines (Figure 4B).

## Discussion

*Phyllomedusa hypochondrialis* represents a well-studied frog species with respect to its skin secretions, with the Antimicrobial Peptide Database listing at least 36 AMPs that have been isolated from this species. Our study identified another novel AMP for the species, with the mature peptide, DLP-PH, First, as the name suggests, showing high identity to the 25-residue B-chain of the AMP distinctin from *Phyllomedusa distincta* (Batista et al., 2001), which itself is unusual among AMPs because of its heterodimeric structure. Furthermore, DLP-PH possessed an intriguing structure consisting of an N-terminal amphiphilic region and a C-terminal hydrophilic region. Altogether, these unique characteristics of DLP-PH argue for it representing the prototype of a novel AMP family. Compared to the majority of amphibian AMPs, DLP-PH is unusual because of the extremely heterogeneous properties of its N- and C-terminal domains, with only the former displaying the characteristics typical of amphibian AMPs (i.e., high amphiphilicity and hydrophobicity) and thus seemingly contributing most to the antimicrobial activity of the entire peptide. By contrast, the C-terminal end was hydrophilic, less amphiphilic and, as evidenced by the DLP-PHc peptide that we designed, devoid of any antimicrobial activity on its own. However, some possible synergistic effects between the two domains of DLP-PH might have been observed in that the entire peptide showed stronger activity against the gram-negative bacteria tested than did either domain alone, and notably the N-terminal domain, in isolation (Table 3). By contrast, the N-terminal domain alone showed better activity against the gram-positive *S. aureus* than did the complete peptide.

Based on these results, we hypothesize that the amphiphilic α-helical N-terminal domain is responsible for the actual bioactivity of DLP-PH, probably by membrane lysis via pore formation. However, the importance of the latter mechanism remains uncertain to some degree, as it does for most amphibian AMPs. Although our cytoplasmic leakage assays did indicate cell lysis against all tested organisms, cell lysis for *E. coli* was minimal at the empirically determined MIC (32 μg/mL) for this organism, implying some other mechanism for the observed bioactivity (e.g., translocation of DLP-PH through the non-lethal pores to instead target intracellular structures or interfere with bacterial metabolism or growth cycles). Similarly, the high leakage of cytoplasmic contents that was inferred for *C. albicans* below its observed MIC (64 μg/mL), indicates that cell lysis alone appears to be insufficient for antimicrobial action and that an additional, possibly intracellular, mechanism is also required. Moreover, the antimicrobial potency of DLP-PH was extremely decreased by the biofilm-forming sessile cells, which indicates it failed to inhibit or eradicate microorganism biofilm, whilst DLP-PH actually inhibited the initial attachment of *P. aeruginosa* at a lower concentration than its MIC. Apparently, such interaction is not able to induce either the lethal of bacterial killing or inhibiting the biofilm formation, we therefore assume that DLP-PH may further interfere the attachment in a non-lethal mechanism, which was reported that cationic AMPs may bind to bacteria cell or cover the non-living surface to reduce bacterial adhesion (Segev-Zarko et al., 2015). In addition, this effect is speculated to be mainly related to the interaction of hydrophobicity instead of charge (Phillips et al., 2007). It also indicates that this effect would be only effective at the early stage of biofilm formation (within 1 h) due to the limited bacterial population, and it would be eliminated by the reproduction of bacteria or generating more hydrophobic compounds. Once the mature biofilm is formed, the antimicrobial action of DLP-PH may be hampered by the neutralization of extracellular polymeric substances (EPS) of biofilm (Batoni et al., 2016).

By contrast, the highly cationic-charged C-terminal domain would appear to be non-obligatory with respect to actual bioactivity, but could facilitate the initial attachment of DLP-PH onto the negatively charged bacterial cell membrane through electrostatic attraction. As we known, the cell membrane of most bacteria consists of more negatively-charged lipids, such as cardiolipin and phosphatidylglycerol, than phosphatidylcholine, and phosphatidylethanolamine that generally constitutes mammalian cell membrane (Yeaman and Yount, 2003). Evidence for the electrostatic attraction comes from molecular docking model, which clearly showed that the cationic charged residues, especially those in the C-terminal domain, mostly faced the negatively charged phospholipid head groups of the bacteria model membrane, employing *E. coli* as a represent. Additionally, the loss of function of DLP-PH in the divalent cation supplemented medium indicates inhibition of the electrostatic attraction to the microbial membrane through competition from the additional cations. This proposed function of the C-terminal domain might also explain why DLP-PH was able to inhibit the growth of at least two human cancer cells without showing other significant cytotoxic effects given that the cell membranes of cancer cells are known to be more negatively charged than those of normal cells (Gaspar et al., 2013). Again, it is unclear if anti-cancer activity results solely primarily cellular membrane lysis (Papo and Shai, 2005) and/or if interacting with cell surface receptors and influencing intercellular signaling pathways (Scott et al., 2000) are also involved. For example, gene microarray studies indicate that some cationic peptides can regulate the expression of a number of genes related to cell apoptosis and cell proliferation (Scott et al., 2000).

Through its heterogeneous nature, DLP-PH structurally somewhat resembles the AMP distinctin, which was isolated from the congeneric frog *Phyllomedusa distincta* (Batista et al., 2001). Distinctin itself is unusual in being a disulphide-bridged, heterodimeric AMP. AMPs with disulphide bridges are known and include human beta-defensins (HBDs) from human leukocytes and epithelial cells (Schneider et al., 2005) and cysteine-rich peptides (CRPs) from plants (Tam et al., 2015), both of which adopt β-sheet structures that are stabilized by two or more intramolecular disulphide bonds. They are also present in the skin secretions of frogs, such as some AMPs from *Rana* spp., which contain a disulphide-bridged cyclic domain of varying size (six or seven residues; the “Rana box” Conlon, 2011) and the Bowman-Birk inhibitor (BBI) like peptides, which contain a canonical disulphide BBI loop with 11 residues (Song et al., 2008). However, in all these cases, the disulphide bridge is intramolecular, whereas the bridge in distinctin is found between the heterodimers, with each chain being encoded by separate mRNAs (Evaristo et al., 2013) as well as having distinct physiochemical properties, albeit not as extreme as we found for the two domains of DLP-PH. The resemblance between DLP-PH and distinctin is further strengthened by the extremely high degree of similarity between the primary structures of the N-terminal domain of DLP-PH and the B-chain of distinctin. However, the exact relationship between the two peptides remains unclear given the complete lack of similarity between the remainder of DLP-PH and any part of distinctin.

A functional similarity between DLP-PH and distinctin also exists insofar as distinctin shows a broad-spectrum antimicrobial activity against both gram-positive and gram-negative bacteria and a generally doubled potency compared to the B-chain in isolation (Table 5). In this, distinctin was more broad-spectrum than DLP-PH, which showed less efficacy against the gram-positive *S. aureus*; however, the antimicrobial activity of DLP-PH against gram-negative bacteria was as good or even better than distinctin or Pexiganan, an antibiotic in late stage clinical trial that is derived from a frog skin AMP. Notably, all AMPs or AMP-derived products were much more effective against the gram-negative bacteria tested than was the conventional antibiotic ampicillin. Considering that DLP-PH is more potent against *E. coli* at the effective concentration without damaging erythrocytes, it might benefit the treatment for *E. coli* induced bacteremia. Additionally, most AMPs process the amphipathic secondary structure, and such structural characteristics have shown the ability of neutralizing a board range of bacteria toxins (Kudryashova et al., 2017), therefore, the further investigation on the inactivation of *E. coli* toxins, like Shiga toxin, by DLP-PH might be interesting in the drug discovery in the treatment of severe toxins-related symptoms.

**Table 5:** Comparative study of the antimicrobial activities of DLP-PH, distinctin, the B-chain of distinctin in isolation, Pexiganan and conventional antibiotic ampicillin.

	**MIC**		
	***S. aureus***	***E. coli***	***P. aeruginosa***
DLP-PH^a^	61.0	7.6	15.2
Distinctin^b^	23.4	11.7	23.4
Distinctin, B-chain^b^	43.3	24.7	43.3
Pexiganan^c^	6.5	12.9	12.9
Ampicillin^a^	0.06	45.8	>366.3

*Unit: μM; ND: not determined*.

a *Determined using the type strains mentioned in this study*.

b *MIC_90_ values at which 90% of the isolates tested were inhibited (Dalla Serra et al., 2008)*.

c *Determined using the same type strains mentioned in this study (Ge et al., 1999)*.

## Conclusions

Given the novel structural and heterogenic physiochemical properties of both DLP-PH and distinctin, it is worth investigating both peptides in more detail as potential antimicrobial and anticancer agents. Both peptides show good bioactivity and, in the case of DLP-PH, minimal cytotoxic effects against normal mammalian cells. One important line of investigation would be to elucidate the potentially different roles of the heterogenic domains of each peptide toward the overall bioactivity. Such information could lead to a greater understanding of the general mechanism(s) of antimicrobial action of AMPs in general, thereby helping to direct drug design (e.g., by modifying existing AMPs to become better drug candidates). The anti-biofilm activity of DLP-PH, although weaker than its general antimicrobial activity, is also worthy of further study as a potential new therapeutic direction in the fight against chronic diseases caused by drug-resistant biofilms. Finally, the fact that we discovered DLP-PH, which not only represents a novel AMP, but might also be a prototype peptide of an entirely new AMP family, in an otherwise well-studied frog species argues for the continued investigation of the skin secretions of amphibians to potentially provide many more new leads for the therapeutic application for antibiotic design and development.

## Author contributions

The conception and design of study was conducted by LW, MZ, and TC. The laboratory work and acquisition of data was performed by DW, YG, YT, and YL. The analysis of data was conducted by CM and XX. The manuscript was drafted by YG, DW, YT, and CM, and revised by OB-E, XX, and CS.

### Conflict of interest statement

The authors declare that the research was conducted in the absence of any commercial or financial relationships that could be construed as a potential conflict of interest.
