# Supplementary material for: Discovery of Distinctin-Like-Peptide-PH (DLP-PH) From the Skin Secretion of Phyllomedusa hypochondrialis, a Prototype of a Novel Family of Antimicrobial Peptide
Source: Front Microbiol. 2018 Mar 23;9:541. doi: 10.3389/fmicb.2018.00541 (PMC5876494; doi:10.3389/fmicb.2018.00541)
Supplement: Supplementary file 1 [file DataSheet1.docx]

**
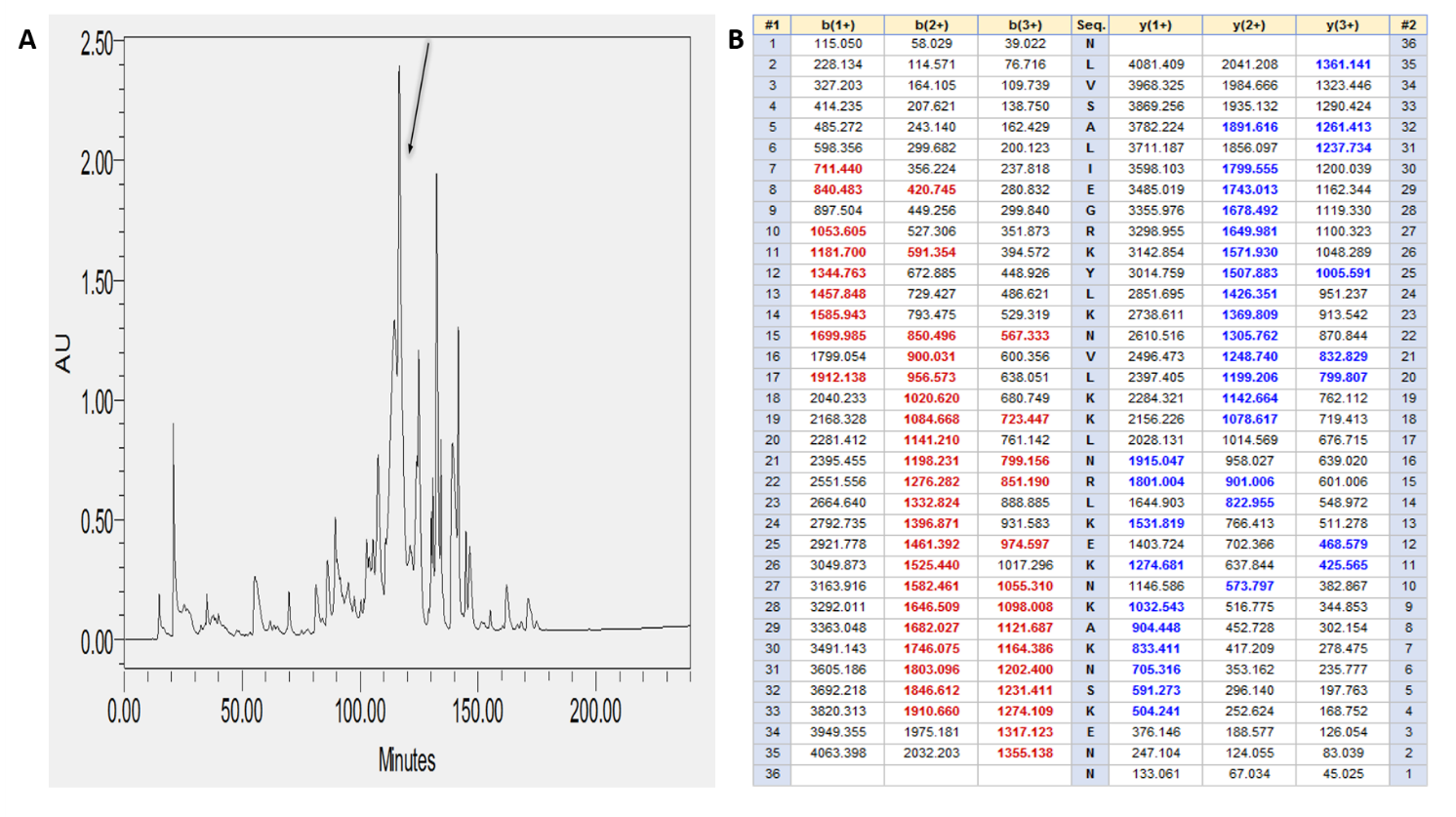
**

**Figure S1. (A)** RP-HPLC chromatogram at 214 nm of *P. hypochondrialis* skin secretion indicating elution position/retention time of DLP-PH (arrow). **(B)** MS/MS fragmentation datasets of fragment ions corresponding to those of DLP-PH. Expected singly- and doubly-charged b-ion and y-ion fragment m/z ratios were predicted and observed fragment ions are indicated in red and blue coloured typefaces.

**Figure S2. (A)** The validation of the predicted DLP-PH 3D model by z-score using ProSA. **(B)** The validation of the predicted DLP-PH 3D model by Ramachandran plot using RAMPAGE. All residues were in the favored regions. **(C)** CD spectra recorded for DLP-PH (100 μM) in 10 mM ammonium acetate/water solution (red) and in 50% 2,2,2-trifluoroethanol (TFE)/10 mM ammonium acetate/water solution (blue). The peptide was existed in random coli in aqueous solution while was induced to a typical α-helix in membrane-mimetic solution.
